# Structural neuroimaging differentiates vulnerability from disease manifestation in colombian families with Huntington's disease

**DOI:** 10.1002/brb3.1343

**Published:** 2019-07-05

**Authors:** Maria del C. Valdés Hernández, Janna Abu‐Hussain, Xinyi Qiu, Josef Priller, Mario Parra Rodríguez, Mariana Pino, Sandra Báez, Agustín Ibáñez

**Affiliations:** ^1^ Department of Neuroimaging Sciences, Centre for Clinical Brain Sciences University of Edinburgh Edinburgh UK; ^2^ Dementia Research Institute University of Edinburgh Edinburgh UK; ^3^ College of Medicine and Veterinary Medicine University of Edinburgh Edinburgh UK; ^4^ Glan Clwyd Hospital North Wales UK; ^5^ Department of Neuropsychiatry Charité–Universitätsmedizin Berlin Berlin Germany; ^6^ School of Psychological Sciences and Health Strathclyde University Glasgow UK; ^7^ Department of Psychology Universidad Autónoma del Caribe Barranquilla Colombia; ^8^ Department of Psychology Universidad de Los Andes Bogotá Colombia; ^9^ Institute of Cognitive and Translational Neuroscience (INCYT) INECO Foundation, Favaloro University Buenos Aires Argentina; ^10^ National Scientific and Technical Research Council (CONICET) Buenos Aires Argentina; ^11^ Centre of Excellence in Cognition and its Disorders Australian Research Council (ARC) Sydney NSW Australia; ^12^ Center for Social and Cognitive Neuroscience (CSCN), School of Psychology Universidad Adolfo Ibáñez Santiago Chile

**Keywords:** basal ganglia, cognition, fluid intelligence, Huntington's Disease, MRI, perivascular spaces, Raven matrices

## Abstract

**Introduction:**

The volume of the striatal structures has been associated with disease progression in individuals with Huntington's disease (HD) from North America, Europe, and Australia. However, it is not known whether the gray matter (GM) volume in the striatum is also sensitive in differentiating vulnerability from disease manifestation in HD families from a South‐American region known to have high incidence of the disease. In addition, the association of enlarged brain perivascular spaces (PVS) with cognitive, behavioral, and motor symptoms of HD is unknown.

**Materials and Methods:**

We have analyzed neuroimaging indicators of global atrophy, PVS burden, and GM tissue volume in the basal ganglia and thalami, in relation to behavioral, motor, and cognitive scores, in 15 HD patients with overt disease manifestation and 14 first‐degree relatives not genetically tested, which represent a vulnerable group, from the region of Magdalena, Colombia.

**Results:**

Poor fluid intelligence as per the Raven's Standard Progressive Matrices was associated with global brain atrophy (*p* = 0.002) and PVS burden (*p ≤* 0.02) in HD patients, where the GM volume in all subcortical structures, with the exception of the right globus pallidus, was associated with motor or cognitive scores. Only the GM volume in the right putamen was associated with envy and MOCA scores (*p* = 0.008 and 0.015 respectively) in first‐degree relatives.

**Conclusion:**

Striatal GM volume, global brain atrophy and PVS burden may serve as differential indicators of disease manifestation in HD. The Raven's Standard Progressive Matrices could be a cognitive test worth to consider in the differentiation of vulnerability versus overt disease in HD.

## 1 INTRODUCTION

Huntington's disease (HD) is a neurodegenerative disorder caused by an expanded Cytosine, Adenine, and Guanine (CAG) repeat on chromosome 4 (Ciarmiello et al., 2017). A formal diagnosis is made when motor symptoms that are typical for HD appear in an individual who has a positive family history of the disease confirmed by gene testing (Tabrizi et al., 2009). As genetic tests can be performed on individuals to identify if they have the expanded CAG repeat, the natural history and pathophysiology of the disease can be studied several years before the disease manifests (Tabrizi et al., 2013). This makes HD a model neurodegenerative disorder to study, which could help understanding other neurodegenerative diseases, which cannot be diagnosed years before onset (Aylward et al., 2013). Classically, HD is characterized by a triad of symptoms including motor, cognitive, and behavioral abnormalities (Baez et al., 2016), and has been associated with neuronal loss within corticostriatal circuits, reflected in brain structural changes visible in magnetic resonance imaging (MRI) (Georgiou‐Karistianis et al., 2013; Kim & Fung, 2014; Lawrence, Sahakian, & Robbins, 1998; McColgan & Tabrizi, 2018). The sensitivity of these volumetric changes in relation to cognitive and social performance, to differentiate manifest HD patients from first‐degree relatives who represent a vulnerable group, would benefit from further documentation, especially across a wide lifespan trajectory and ethnic groups.

Several studies conducted under the framework of international multisite observational projects for the study of HD (e.g., TRACK‐HD, COHORT, PREDICT‐HD), have revealed many clinical and biological indicators of disease progression, including neuroimaging parameters (Aylward, 2014; Aylward et al., 2013). For example, studies have found strong correlations between many gray and white matter regions and clinical tests, including recognition of negative emotions, metronome tapping precision, and measures of tongue force. A study found that the symbol digit modalities test, a measure of processing speed, was correlated with annual whole‐brain volume loss in HD patients (Tabrizi et al., 2011). White matter atrophy has been reported in the earliest premanifest stages of HD (Aylward et al., 2013; Tabrizi et al., 2013). It has also been noted that atrophy of individual brain structures, specifically in the striatum (i.e., accumbens nucleus, caudate nucleus, putamen, and pallidum), could be a more sensitive biomarker compared to whole‐brain volume (van den Bogaard et al., 2011, Tabrizi et al., 2011, 2013). For example, it has been found that cortical gray matter volume and thickness are reduced in premanifest individuals, from 9 to 15 years before the clinical onset of HD (Jurgens et al., 2008). In these preonset stages, volume losses in striatal structures have been associated with higher chorea scores (Coppen, Jacobs, Berg‐Huysmans, Grond, & Roos, 2018; Jurgens et al., 2008), smaller putamen volume with motor deficits, and atrophy in the caudate has been correlated with cognitive decline (Coppen et al., 2018). However, although striatal atrophy is considered a signature of the disease (Aylward, 2014), the suitability of, specifically, gray matter loss in the striatum to differentiate overt HD from vulnerability, regardless of whether the latter are gene carriers or not, would benefit from further analyses involving individuals across different ethnic groups.

Despite notable efforts to document this disease, most of the information available has been provided by studies involving individuals from North America, Australia, and Western Europe. It therefore seems important to validate previous analyses in samples from communities geographically distant and ethnically different from those that usually contribute to data, especially because environmental factors are known to modulate motor, cognitive, affective, and other symptoms of HD (Mo, Hannan, & Renoir, 2015). We attempted to fill this void in the current scientific literature by studying the neuroimaging atrophy markers of HD reported by previous studies in a cohort of HD patients and their first‐degree relatives from Magdalena, Colombia. This Colombian region has a high prevalence of the disease, previously reported as the second largest worldwide (Baez et al., 2015, 2016, 2018; Kargieman et al., 2014). Impairments in negative emotion recognition and empathy for pain (Baez et al., 2015), reduced Schadenfreude (Baez et al., 2016), and motor‐language coupling (Kargieman et al., 2014) have been reported to affect HD families in the region. A study that involved HD patients from this region and age‐matched unrelated controls associated the ventral striatum, precuneus, and superior parietal lobule with Schadenfreude in HD patients (Baez et al., 2018). The atrophy patterns reported by Baez and colleagues, which involved the basal ganglia and thalamus as well as the frontal and parietal cortex, matched previous reports on HD (Baez et al., 2018).

Moreover, the need for reliable markers of disease progression remains an area of intense research (Kim & Fung, 2014). Enlarged perivascular spaces (PVS), a known indicator of brain atrophy and white matter pathology (Wardlaw et al., 2013), are pial‐lined interstitial fluid‐filled spaces in the brain that surround perforating vessels and have been identified a feature of small vessel disease (Wardlaw et al., 2013), playing an important role in sleep disorders (Berezuk et al., 2015). Traditionally, PVS have been considered passive anatomical structures resultant from transient vasoconstriction and vasodilation of the vessels they surround. Their appearance also in the absence of any pathology has perhaps contributed to their neglect in studies of neurodegenerative diseases. However, recent studies have shown they play an active role in cleaning toxic solutes and are a sign of glymphatic fluid stasis, playing a role in the pathogenesis of neurodegenerative diseases (Mestre, Kostrikov, Mehta, & Nedergaard, 2017). Hence, their visibility and size have been reported to increase with age (Francis, Ballerini, & Wardlaw, 2019), in Alzheimer's disease (Ramirez et al., 2015), and they are considered highly heritable (Duperron et al., 2018). Although the presence of PVS in HD patients has not been reported in the scientific literature so far, neuroradiological reports of PVS in HD patients has been a matter of concern for caregivers in one of the internet support forums hosted by the HD Lighthouse Families Community (http://www.hdlf.org/community). In this study, we evaluate, for the first time, whether PVS can be considered a neuroimaging marker that differentiates vulnerability versus overt HD, and their association with cognitive, functional, and behavioral indicators in HD patients and their first‐degree relatives. We hypothesize that the PVS burden will be higher in HD patients compared to their first‐degree relatives. However, as cross‐sectional cognitive studies have not found a strong effect of total PVS burden on general cognition (Huijts et al., 2014; Hurford et al., 2014; Zhu et al., 2010), we did not expect an association of PVS burden with functional, cognitive, or behavioral symptoms of HD.

## 2 MATERIALS AND METHODS

### 2.1 Subjects

This study included 29 individuals from the region of Magdalena, Colombia: 15 patients and 14 first‐degree relatives. The HD patients were diagnosed both genetically and clinically with the disease, and the first‐degree relatives were either descendants or siblings of the patients. Individuals in this family group were not diagnosed with HD when the study was conducted. Although relatives did not receive genetic testing, this population represents a vulnerability group. Biological (Markianos, Panas, Kalfakis, & Vassilopoulos, 2008), clinical (Dorsey, 2012), and cognitive (Baez et al., 2015; Giordani et al., 1995; Kargieman et al., 2014) factors of familial vulnerability have been reported irrespective of whether the first‐degree relatives are HD mutation carriers or not. Vulnerability to HD means that individuals with a family history of the disease have a high probability of developing it or some unspecific deficits related to it. Assessing these individuals is important to understand the nature of HD and identify potential HD biomarkers (Baez et al., 2015, 2016; Kargieman et al., 2014).

Both groups were assessed using the Unified Huntington's Disease Rating Scale (UHDRS) (Siesling, Vugt, Zwinderman, Kieburtz, & Roos, 1998). The mean UHDRS score reported for patients was 20.5 (*SD* 8.6), and for relatives it was 0.2 (*SD* 0.4) (Baez et al., 2015). In addition, HD patients were assessed with the Total Functional Capacity Scale (HDFCS) (Shoulson & Fahn, 1979). The mean HDFCS score reported was 11.8 (*SD* 1.5) (Baez et al., 2015). Thirteen patients (87%) did not receive any pharmacologic treatment. Two patients (13%) were taking antidepressants, antipsychotics, or/and Tetrabenzine. Patients and relatives had no history of other major neurological illness, psychiatric disorders, or alcohol/drug abuse. All participants completed written informed consent, and the ethics committee of the Institute of Cognitive Neurology and the Autonomous Caribbean University approved the study (resolution 59‐A) and the use of the data for research purposes (Baez et al., 2016).

### 2.2 MRI acquisition

The brain MRI scans were obtained from a 1.5T Siemens Magnetom scanner equipped with a standard head coil, and were made available for this study after a full anonymization using Dicom Confidential (https://sourceforge.net/projects/privacyguard/), an open‐source java DICOM de‐identification tool. This study analyzed the T1‐weighted, Fluid Attenuated Inversion Recovery (FLAIR), and T2‐weighted MRI. The 3D T1‐weighted (gradient echo inversion recovery) sequence had TR/TE/TI = 2070/5.18/1100 ms, flip angle 15^o^, voxel size [0.9375, 0.9375, 1], acquisition matrix 256x256x176. The T2‐weighted (spin echo) sequence was acquired in axial orientation, with TR/TE = 4,860/110 ms, slice thickness 5mm, voxel size [0.4492, 0.4492, 6.5], acquisition matrix 418x512x20. The FLAIR (spin echo inversion recovery) sequence was also acquired axially, with TR/TE/TI = 11,000/140/2500 ms, voxel size [0.8984, 0.8984, 6.5], acquisition matrix 232 × 256 × 20.

### 2.3 Image analysis

#### 2.3.1 Volumetric measurements

Contents of intracranial volume (ICV) within the inner skull table including normal appearing white matter, cerebrospinal fluid, brain tissue, veins and dura, were initially extracted automatically using the brain extraction tool (BET2) from the FMRIB software library (FSL) (https://fsl.fmrib.ox.ac.uk/fsl/fslwiki). Binary ICV masks were visually checked and manually edited in axial, coronal, and sagittal planes when required using Mango (http://ric.uthscsa.edu/mango/). Normal appearing white matter (NAWM) and cerebrospinal fluid (CSF) were also segmented using another tool from the same library: the FSL automatic segmentation tool (FAST). The structures of the basal ganglia and thalami were extracted using a combination of other three tools also from the same library: Smallest Univalue Segment Assimilating Nucleus (SUSAN), FMRIB's Linear Image Registration Tool (FLIRT) and a model‐based segmentation/registration tool (FIRST), combined in an automatic pipeline developed in‐house (Valdés Hernández et al., 2015). The probabilistic maps resultant from the tissue extraction was combined with the results from the subcortical segmentations to discern the volume of gray matter (GM) tissue in the striatal structures. These results were visually assessed for further manual editing when it was required. In addition, for compatibility with other studies that had calculated the full volume of the subcortical structures, the latter were also calculated. Figure S1 in the Supplementary material illustrates the differences between the GM tissue (i.e., volumes analyzed here) and the boundaries of the whole right and left putamen structures in one study participant.

PVs were segmented automatically using a MATLAB Graphic Unit Interface “Extracting_small_T2W_hyperintensities,” part of the library of MATLAB tools bric1936 freely available from https://sourceforge.net/projects/bric1936/files/MATLAB_R2015a_to_R2017b/. This GUI performs an adaptive histogram equalization to enhance the contrast between hyper‐/hypo‐intensities and background, to automatically extract signal‐enhanced features from the background or tissue. It also uses connected component analysis to constraint the size of these features and describe their properties. This GUI also allows to manually remove false positives and to change the sensitivity of the detection algorithm. Finally, it outputs volume and count of each structure identified per slice. Total PVS volume and count were used in our analyses.

All brain volumes were adjusted for head size related differences using ICV. All measurements were performed blind to any other clinical, cognitive, or demographic information. Three observers of different levels of experience (JA, XQ, and MVH) assessed each segmentation result, and discrepancies were discussed until a final agreement was reached.

#### 2.3.2 Visual PVS rating

Despite recent computational methods for assessing PVS have shown promising for being applied in clinical research and practice (Ballerini et al., 2018), still the gold‐standard method for assessing PVS is neuroradiological (i.e., visual) rating. A trained observer (XQ) rated the PVS using the Potter scale (Potter, Chappell, Morris, & Wardlaw, 2015). Briefly, this scale rates the PVS separately in three major anatomical brain regions: midbrain, basal ganglia and centrum semiovale, in T2‐weighted axial slices representative of the PVS burden in each region. The rating is done separately for left and right hemispheres, but a combined score that represents the average of the PVS burden in each region is given. The rating can be 0 (no PVS), 1 (mild; 1–10 PVS), 2 (moderate; 11–20 PVS), 3 (frequent; 21–40 PVS), or 4 (severe; >40 PVS) (Potter et al., 2015). Ratings were done twice by the same rater, blind to any clinical, demographic, or cognitive information, and to the previous scores. Where intra‐observer differences were observed, the final score was decided on a team discussion of each case, also blind to any other information.

### 2.4 Functional assessments

The Myer's functional score was used to assess the level of disability. This score ranges from 100 to 10, with a score of 10 indicating high levels of disability. This physical disability score has a high inter‐rater reliability (Myers et al., 1991).

### 2.5 Cognitive assessments

The general cognitive status was assessed with the Montreal Cognitive Assessment (MOCA). MOCA is a very sensitive cognitive screening tool that covers important cognitive domains such as short‐term memory, working memory, language, and orientation (Nasreddine et al., 2005). To assess executive function, the INECO frontal screening (IFS) test was used. It includes eight subtests; motor programming, conflicting instructions, motor inhibitory control, numerical working memory, verbal working memory, spatial working memory, abstraction capacity, and verbal inhibitory control (Torralva, Roca, Gleichgerrcht, Lopez, & Manes, 2009). To assess fluid intelligence, the Raven's Standard Progressive Matrices were used. This 60‐item test assesses fluid intelligence nonverbally, and is often included in research on patients with cognitive deficits, however, has only rarely been used in patients with HD (Bilker et al., 2012).

### 2.6 Socio‐emotional assessments

A social cognition test indexing social emotions was performed by all study participants. In it, each participant was shown a real‐life photograph and a description of two characters matched in age and gender with the participant.

The test comprised two experimental blocks (i.e., parts A and B), and it was performed using a battery administered by the Data Host Institution in Colombia. Part A investigated envy ratings. In it, participants read eight sentences describing four fortunate events involving each character. After reading each sentence, participants rated the event in terms of how much envy they felt for the character (1 = no envy, 9 = extreme envy). Part B calculated the Schadenfreude ratings. In it, participants read and reported the intensity of their pleasure (i.e., schadenfreude: 1 = no pleasure, 9 = extreme pleasure) in response to eight unfortunate events (e.g., cuckoldry caused by the character's girlfriend/boyfriend) happening to the characters (i.e., also four events per character). Two neutral events (e.g., washing clothes) were included in each experimental block. Events were presented in pseudo‐randomized order. These socio‐emotional scores have been previously validated in HD (Baez et al., 2016, 2018).

### 2.7 Statistical analyses

All brain volume variables were standardized by ICV and expressed as percentage of it. Statistical analyses were performed using IBM SPSS Statistics 21.0.0. The sample was divided into two groups: the “patient group” and the “family group,” the latter grouping the first‐degree relatives. As one of our main goals was to investigate whether the imaging markers analyzed were sensitive enough to be considered neuroimaging markers that differentiate vulnerability from disease manifestation in HD families, both study groups were assessed separately in cross‐correlation analyses and linear regression models. Bivariate cross‐correlations were performed with bootstrap. Age, sex, and years of education were used as covariates in the univariate linear regression models to control for any confounding effects these variables may have. In these models, our dependent variable was either the functional, cognitive, or socio‐emotional score and the independent variable was the brain volumetric (or score) variable. Bootstrap was also used in linear regression models, which were double‐checked against general linear models. Both groups were compared using the Median and Mann‐Whitney U tests to find parameters that differ significantly between them.

## 3 RESULTS

### 3.1 Sample characteristics

The descriptive characteristics of the imaging and cognitive variables involved in the analyses are given in Table 1. The mean age of the total sample was 36.76 years (HD patients: 45.87, standard deviation (*SD*) 9.42 years old and family: 27 (*SD* 7.89) years old). The descriptive statistics of each sex subgroup for relatives and patients appears in the Table S1. On average, female participants had one year more of education than males in the patient group and the opposite pattern in the family group. The group of HD patients (*n* = 15, eight female) was on average approximately 20 years older and had 3 fewer years of education than the family group (*n* = 14, 11 female). Age and years of education significantly differed between family and patient groups.

**Table 1:** Descriptive statistics of the variables involved in the analyses

	Descriptive statistics		Group comparison (*p*‐values)		Total (mean ± *SD*)
	HD Patients (*n* = 15) (mean ± *SD* or median (IQR))	First‐Degree Relatives (*n* = 14) (mean ± *SD* or median (IQR))	Median test	Mann‐Whitney U test	
Demographic Variables (years)					
Age	45.87 ± 9.42	26.38 ± 7.86	0.001	0.007	36.76 ± 12.86
Education	9.20 ± 3.19	12.77 ± 2.17	0.001	0.001	10.97 ± 3.26
Imaging Volumetric Variables (% in ICV)					
NAWM	35.70 ± 1.67	38.063 ± 1.63	0.13	0.0040	36.80 ± 2.017
CSF	28.30 ± 3.11	19.67 ± 2.38	<0.0001	<0.0001	24.29 ± 5.17
Left caudate nucleus	0.11 ± 0.041	0.20 ± 0.037	<0.0001	<0.0001	0.15 ± 0.061
Right caudate nucleus	0.094 ± 0.060	0.21 ± 0.037	<0.0001	<0.0001	0.15 ± 0.078
Left putamen	0.075 ± 0.028	0.14 ± 0.035	<0.0001	<0.0001	0.11 ± 0.046
Right putamen	0.071 ± 0.024	0.16 ± 0.038	<0.0001	<0.0001	0.11 ± 0.053
Left globus pallidus	0.0066 ± 0.0053	0.0070 ± 0.0037	1.00	0.62	0.0068 ± 0.0041
Right globus pallidus	0.0052 ± 0.0033	0.0075 ± 0.0047	1.00	0.29	0.0063 ± 0.0041
Left thalamus	0.16 ± 0.029	0.21 ± 0.032	0.0020	<0.0001	0.18 ± 0.040
Right thalamus	0.17 ± 0.023	0.22 ± 0.025	0.0020	<0.0001	0.19 ± 0.036
BGPVS	0.030 ± 0.017	0.021 ± 0.0084	0.45	0.16	0.026 ± 0.015
PVS visual scores (Potter scale) [median (IQR)]					
Basal GANGLIA	1 (1)	1 (0)	0.0070	0.037	1 (1)
Centrum SEMIOVALE	2 (1)	1 (1)	0.14	0.12	1 (1)
Midbrain	0 (1)	0 (1)	0.71	0.68	0 (1)
Functional, Cognitive and Socio‐emotional Assessment Scores (arbitrary units)					
Myer's Functional Score	76.77 ± 14.47	99.23 ± 2.77	—	<0.0001	87.59 ± 15.51
IFS_Total	14.53 ± 5.90	22.23 ± 2.95	0.0030	<0.0001	18.17 ± 6.00
MOCA_Total	17.07 ± 4.68	26.15 ± 2.85	<0.0001	<0.0001	21.24 ± 5.92
Raven Matrices	4.67 ± 4.22	12.08 ± 6.59	0.0090	0.001	7.97 ± 6.46
Soc.Emot. Part A Total	30.67 ± 20.15	36.77 ± 16.92	0.72	0.19	34.41 ± 18.95
Soc Emot. Part B Total	44.40 ± 19.26	23.38 ± 12.66	0.0080	0.0010	33.90 ± 19.50

Note

PVS visual scores values are median and interquartile range (nonparametric ordinal variables).

Abbreviations: NAWM: normal appearing white matter; CSF: cerebrospinal fluid; BGPVS: basal ganglia perivascular spaces.

### 3.2 Group comparison

**Figure 1:**
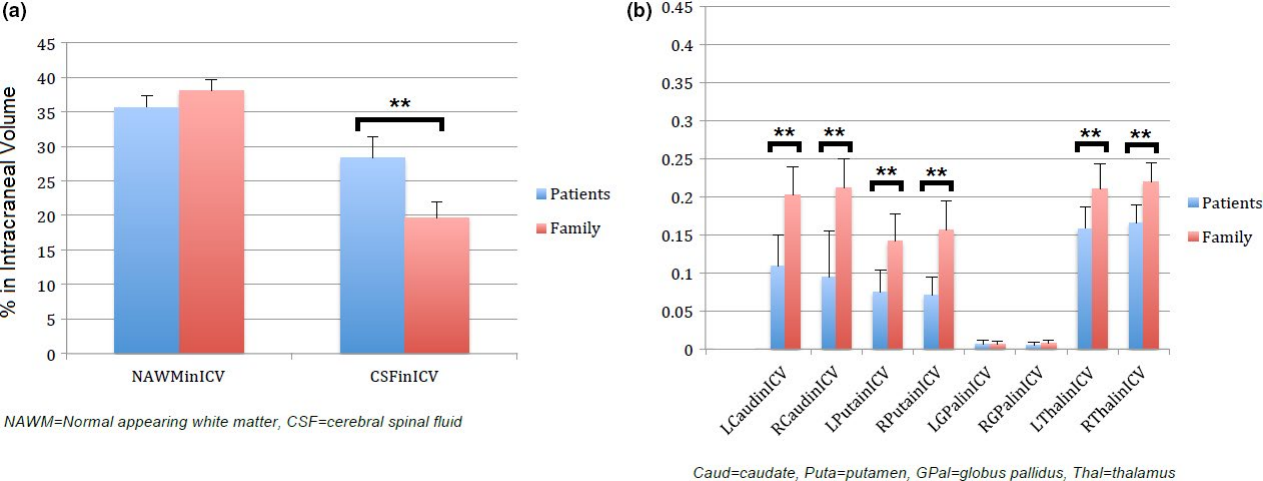
Illustration of the magnitude of the volumetric measurements per group. (a) Global atrophy‐related measurements and (b) gray matter volume in the subcortical structures analyzed. Significance was determined without adjusting for any demographic variable (i.e., age, biological sex)

**Figure 2:**
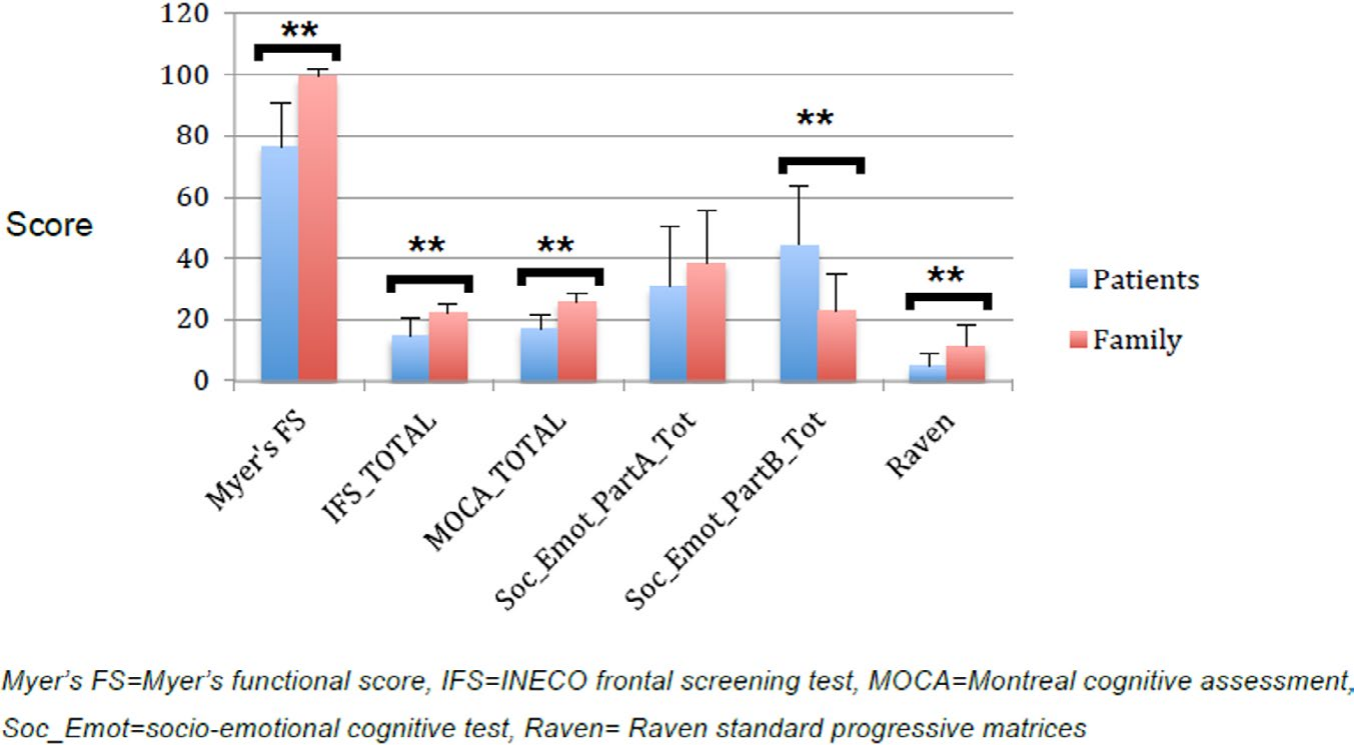
Illustration of the magnitude of the cognitive, motor, and behavioral scores per group. Significance was determined without adjusting for any demographic variable (i.e., age, biological sex)

The significance values obtained from the Median and Mann–Whitney *U* tests are shown in Table 1. Figures 1 and 2 illustrate the magnitudes of the brain volumetric measurements and the functional, cognitive and socio‐emotional tests scores respectively, in each group. The median values of NAWM, left, and right GM in the globus pallidus and basal ganglia PVS volumes, were not significantly different between the family and patient groups. The median and distribution of the rest of the volumetric variables and the visual score of PVS burden in the basal ganglia differed between both groups (See Table 1). The Myer's functional score differed between family and patient groups (*p* < 0.0001). The scores from the three cognitive tests were significantly different between groups (*p* < 0.001). The scores from the socio‐emotional test part B (Schadenfreude) differed between groups (*p* < 0.001), but not those from the part A (envy) (*p* > 0.15).

### 3.3 Bivariate relations in each group

Table 2 shows the nonparametric bootstrapped bivariate relations of the functional, cognitive, and socio‐emotional tests results with the neuroimaging assessments (volumetric, adjusted by head size, and visual ratings), for the imaging variables that were correlated with at least one of the functional, cognitive, or socio‐emotional scores, in each group. Parametric (Pearson's) correlations yielded similar results.

**Table 2:** Spearman (ρ) correlation relations of the functional, cognitive, and socio‐emotional tests results with the neuroimaging assessments (volumetric, adjusted by head size, and visual ratings), for the imaging variables that were correlated with at least one of the functional, cognitive or socio‐emotional scores, in each group

Group	Imaging variables	Functional scores	Cognitive scores			Socio‐emotional scores	
		Myer's Functional Score	IFS (total)	MOCA (total)	Raven matrices	Socio‐emotional Part A (total)	Socio‐emotional Part B (total)
Patients (*n* = 15)	CSF volume in ICV	−0.567*	−0.473	−0.314	−0.255	−0.310	0.098
	Left Caudate volume in ICV	0.569*	0.568*	0.583*	0.165	0.236	−0.209
	Right Caudate volume in ICV	0.332	0.593*	0.531*	−0.049	0.312	−0.308
	Left Putamen volume In ICV	0.539*	0.474	0.353	−0.047	0.319	−0.05
	Right Putamen volume in ICV	0.528*	0.589*	0.583*	0.091	0.328	−0.285
	Right Globus Pallidus volume in ICV	0.543*	0.294	0.325	0.000	0.389	0.038
	Left Thalamus volume in ICV	0.308	0.084	−0.023	−0.016	0.748**	−0.360
	Basal ganglia PVS score	−0.662**	−0.403	−0.233	−0.204	−0.047	−0.047
	Centrum semiovale PVS score	−0.605*	−0.120	0.000	0.264	−0.507	0.023
Family (*n* = 14)	Left Caudate vol. in ICV	−0.386	−0.082	0.090	0.640*	0.105	−0.290
	Centrum semiovale PVS score	−0.527	−0.227	−0.200	0.245	0.098	−0.606*

Abbreviations: CSF: cerebrospinal fluid; ICV: intracranial volume; PVS: perivascular spaces; NAWM: normal‐appearing white matter; IFS: INECO Functional Screening test; MOCA: Montreal Cognitive Assessment.

* *p* < 0.05.

** *p* < 0.01 both 2‐tailed.

#### 3.3.1 Correlations between the imaging variables and the functional score

The Myer's functional scores were only correlated with variables in the patient group but not in the family group. In patients, the GM volumes of the left caudate, left and right putamen, right globus pallidus, and general brain atrophy correlated with the Myer's scores (*p* < 0.05). However, the only variable that had a strong and significant correlation (Spearman ρ = −0.662, *p* = 0.007) with the functional scores was the PVS visual score in the basal ganglia. The PVS scores in the centrum semiovale correlated with the Myer's tests scores at the *p* < 0.05 significance level only in the patient group (Spearman ρ = −0.605, *p* = 0.017).

#### 3.3.2 Correlations between the imaging variables and the cognitive scores

The outcome from the INECO frontal screening and MOCA tests correlated with the left/right caudate and right putaminal GM tissue volumes only in the patient group (*p* < 0.05). The volume of GM tissue in the left caudate was correlated with the Raven matrices results only in the family group (*p* < 0.05).

#### 3.3.3 Correlations between the imaging variables and the socio‐emotional test scores

The outcome from the socio‐emotional test part A (i.e., envy ratings) correlated strongly and significantly with the volume of the GM tissue in the left thalamus of patients (Spearman ρ = 0.748, *p* = 0.001) but not in first‐degree relatives. The outcome from the socio‐emotional test part B (i.e., Schadenfreude ratings) correlated with the PVS scores in the centrum semiovale in the family group (i.e., not in patients) at the *p* < 0.05 level of significance (Spearman ρ = −0.606, *p* = 0.028).

### 3.4 Associations in each group

Table 3 shows the values of the nonstandardized coefficients, standard errors, and significances in the linear regression models.

**Table 3:** General linear models parameters estimates (Significance at *p* < 0.05 is indicated in bold). Covariates are age, biological sex, and years of education

Group	Dependent variable in the models	Independent variable in the models					
	Imaging variable	Functional scores	Cognitive scores			Socio‐emotional scores	
		Myers	IFS total	MOCA total	Raven Matrices	Socio‐emotional (Part A) total	Socio‐emotional (Part B) total
Patients (*n* = 15)	CSF volume in ICV	**−3.49; 1.52; 0.045**	−0.32; 0.65; 0.63	−0.45; 0.51; 0.40	**−0.93; 0.23; 0.002**	−0.45; 2.089; 0.83	0.57; 2.39; 0.81
	Left Caudate vol. in ICV	**256.95; 98.28; 0.026**	31.17; 43.33; 0.49	30.11; 34.72; 0.41	16.98; 24.57; 0.51	156.95; 133.019; 0.26	−34.41; 162.37; 0.21
	Right Caudate vol. in ICV	104.82; 92.36; 0.28	**64.81; 27.37; 0.039**	**53.14; 22.028; 0.037**	3.33; 19.32; 0.87	14.027; 109.16; 0.13	−105.087; 120.74; 0.41
	Left Putamen vol. in ICV	**459.36; 183.52; 0.031**	42.23; 80.46; 0.61	55.81; 63.69; 0.40	**89.32; 36.51; 0.034**	−10.96; 260.56; 0.97	−67.007; 297.93; 0.83
	Right Putamen vol. in ICV	**472.45; 217.82; 0.055**	87.23; 87.81; 0.34	124.24; 63.39; 0.078	**94.31; 42.71; 0.052**	79.53; 293.00; 0.27	−238.18; 328.55; 0.48
	Right Globus Pal. vol. in ICV	2024.78; 1545.57; 0.22	9.14; 582.97; 0.99	414.13; 453.85; 0.38	420.32; 301.98; 0.19	1931.50; 1759.60; 0.30	133.16; 2,134.52; 0.95
	Left Thalamus vol. in ICV	252.36; 218.35; 0.27	−45.89; 79.70; 0.58	−4.42; 65.63; 0.95	**86.58; 36.77; 0.040**	**576.46; 183.75; 0.011**	−365.53; 273.23; 0.21
	Basal Ganglia PVS score	**−18.87; 7.39; 0.029**	−3.64; 3.11; 0.27	−2.84; 2.53; 0.29	**−3.85; 1.42; 0.022**	3.29; 10.53; 0.76	−2.25; 12.10; 0.86
	Centrum Semiovale PVS score	−12.36; 6.03; 0.067	0.92; 2.49; 0.72	−0.27; 2.027; 0.90	**−3.056; 1.037; 0.015**	−7.57; 7.63; 0.34	5.029; 9.031; 0.59
	Midbrain PVS score	−5.55; 9.77; 0.58	−0.77; 3.45; 0.83	−2.13; 2.72; 0.45	**−4.070; 1.48; 0.020**	7.58; 10.790; 0.50	3.89; 12.61; 0.76
Family (*n* = 14)	NAWM volume in ICV	0.18; 0.690; 0.80	0.48; 0.44; 0.30	0.47; 0.67; 0.50	0.34; 1.13; 0.78	**−6.41; 2.54; 0.036**	−0.42; 2.35; 0.86
	Left Caudate vol. in ICV	−29.48; 29.42; 0.35	−8.43; 21.001; 0.70	22.015; 30.26; 0.49	86.11; 41.20; 0.070	152.71; 143.54; 0.32	90.68; 101.079; 0.40
	Right Putamen vol. in ICV	16.63; 33.17; 0.63	−20.23; 21.74; 0.38	**−70.39; 22.75; 0.015**	−34.82; 53.90; 0.54	**364.73; 103.74; 0.008**	−116.18; 106.81; 0.31
	Centrum Semiovale PVS score	−3.43; 2.016; 0.13	−0.088; 1.60; 0.96	−0.48; 2.35; 0.84	2.91; 3.72; 0.46	1.27; 11.55; 0.92	−8.56; 7.39; 0.28

Abbreviations: CSF: cerebrospinal fluid; ICV: intracranial volume; PVS: perivascular spaces; NAWM: normal‐appearing white matter; IFS: INECO Functional Screening test; MOCA: Montreal Cognitive Assessment.

#### 3.4.1 Associations between the imaging variables and the functional scores

The Myer's functional scores were only associated with imaging parameters in the patient group, but not in the family group. From the imaging variables that were correlated with the functional score, only the volume of the GM tissue (i.e., excluding white matter and vascular abnormalities) in the right globus pallidus and the PVS score in the centrum semiovale were not associated with the functional score after correcting for age, biological sex, and years of education. Of note, the significance in the associations that held (i.e., with global atrophy, left caudate, right/left putamen GM volumes, and basal ganglia PVS scores), the level of significance was *p* < 0.05.

#### 3.4.2 Associations between the imaging variables and the cognitive scores

Accounting for age, biological sex, and years of education changed the pattern of bivariate correlations between imaging and cognitive variables. Only in the patient group (and not in the family group), the outcome of the cognitive tests was associated with some of the imaging variables analyzed. Fluid intelligence (i.e., from Raven matrices) was associated with global atrophy (β = −6.84, *p* = 0.002), left putaminal GM tissue volume (β = 0.600, *p* = 0.034), GM tissue volume in the left thalamus (β = 0.592, *p* = 0.040), and PVS scores in all regions: basal ganglia (β=−0.471, *p* = 0.022), centrum semiovale (β = −0.578, *p* = 0.015), and midbrain (β = −0.489, *p* = 0.020). The INECO frontal screening and MOCA scores only were associated with the right caudate GM tissue volume in patients (β = 0.662, *p* = 0.039 and β = 0.684, *p* = 0.037 respectively). MOCA scores were associated with the right putaminal GM tissue volume in the family group (β = −0.948, *p* = 0.015).

#### 3.4.3 Associations between the imaging variables and the socio‐emotional scores

Only the socio‐emotional test part A (i.e., envy ratings) was associated with imaging variables after controlling for age, biological sex, and years of education. In the patient group, it was associated with the GM tissue volume in the left thalamus (β = 0.825, *p* = 0.011), and in the family group it was associated with the volume of total NAWM (β = −0.618, *p* = 0.036), and with the GM tissue volume in the right putamen (β = 0.829, *p* = 0.008).

### 3.5 Comparability with published outcomes

Volumetric differences between the conventionally reported subcortical volumes and the volumes of the GM tissue in these structures (i.e., measurements analyzed and reported in this study) exist. As Figure S2 and Table S2 show, subcortical volumes were, on average 20% bigger (confidence interval also 20%) than the GM volume in these structures, and these differences were slightly higher in higher volumes. After adjusting for intracranial volume (i.e., head size), the differences between these measurements were 1% smaller on average. The globus pallidus was, however, generally two times bigger than the GM volume in this structure.

## 4 DISCUSSION

### 4.1 Main findings

In line with our main hypotheses, the GM volume in the basal ganglia and thalami resulted a sensitive differential indicator of disease manifestation versus vulnerability in HD families from a South‐American region known to have high incidence of the disease. In addition, we examined for the first time the PVS burden in HD patients compared to first‐degree relatives, and found that the PVS burden was predictive of reduced fluid intelligence in HD patients only, and not in the vulnerable group, opening a new area of research. As result of our analyses, from all cognitive and socio‐emotional tests applied, the Raven's Standard Progressive Matrices exhibited the greatest discriminatory power for overt HD versus vulnerability to the disease after adjusting for age, biological sex, and years of education; thus suggesting its usefulness in the study of this disease.

Our finding that whole‐brain atrophy (i.e., for which CSF volume is a surrogate measure) was associated with poorer performance in the Raven test in HD patients, is in agreement with previous studies that have also found whole‐brain volume loss to be associated with cognitive measures in HD patients (Tabrizi et al., 2011). Whole‐brain atrophy is not just present in manifest HD stages, but also in premanifest stages, as it can appear as much as 10 years before predicted onset (Tabrizi et al., 2009). The fact that both the left and right putaminal GM volumes were associated with Raven test results in HD patients is in line with a previous study by Jurgens et al. (2008) who found a link between smaller putamen volume and cognition in patients with premanifest HD, specifically in the Trail Making Test B and symbol digit modalities tests, although did not consider any confounding factor. Aylward et al. (2013) also observed that structures in the basal ganglia contributed more than other brain regions to the prediction of cognitive performance in individuals with prodromal HD. Cross‐sectional studies have found that reductions in putaminal volume can appear up to 15–20 years before disease onset in carriers of the mutant allele (Scahill, Andre, Tabrizi, & Aylward, 2017). The association we observed between the Raven matrices scores and the GM volumes of the left thalamus in HD patients has not been previously reported. Kassubek, Juengling, Ecker, and Landwehrmeyer (2005) found correlations between thalamic volume and cognition in individuals with HD. They reported that the thalamic subnuclei projecting to prefrontal areas and connected to the striatum particularly displayed volume loss. Recent studies suggested that the thalamus only shows significant atrophy in the later stages of HD (Hobbs et al., 2010; Scahill et al., 2017). Thus, the negative association between fluid intelligence and PVS burden we observed in HD patients warrants further research.

The socio‐emotional test part A was also associated with the GM volume in the left thalamus in HD patients but not in the vulnerable group. The effects of thalamic GM volume on socio‐emotional cognition in HD have not been investigated. This is surprising as the thalamus is part of the limbic system, which is largely known for its associations with emotion processing (Wilkos, Brown, Slawinska, & Kucharska, 2015). The important role of the thalamus in the brain network involved in emotion processing has been highlighted by several studies (Pessoa, 2017). A study on neural correlates of jealousy identified the thalamus as one of the structures involved (Sun et al., 2015). It has been proposed that the neural correlate of envy involves the thalamus in the process of response to emotions, which are projected to the insula, and then into the orbitofrontal and anterior cingulate regions of the frontal lobe (Kong, 2010). Moreover, associations between thalamic volume and socio‐emotional cognition have been reported in stroke patients with thalamic lesions from a stroke (Liebermann et al., 2013). The thalamus and basal ganglia are part of parallel segregated circuits with cortical areas such as the dorsolateral prefrontal cortex, the lateral orbitofrontal cortex, and the anterior cingulate/medial orbitofrontal cortices (Bonelli & Cummings, 2007). These three regions contribute not only to emotion regulation but also to planning working memory, rule‐based learning and attention (Bonelli & Cummings, 2007). This may explain why we found that the GM volume of the left thalamus and putamen were associated with certain aspects of cognition, tested by the Raven matrices test and the socio‐emotional test part A, as atrophy in these regions surely affects the circuits that control cognitive, behavioral and emotional functions (Cheung et al., 2006).

The caudate is part of the dorsolateral prefrontal cortex circuit, which plays a part in modulating the cognitive‐control process and suppresses unwanted behavior, which is essential for cognitive control (Aylward et al., 2013; Misiura et al., 2017). We therefore expected associations between caudate GM volumes and the outcome of the IFS and MOCA tests in the patient group. Several other studies have found similar associations. For example, Misiura et al. (2017) found that performances on the symbol digit modalities test, an emotion recognition test, and a task requiring internal timing, were associated with caudate volume in premanifest HD. It has also been found that the Mini Mental State Examination (MMSE) scores correlate with caudate volume in the early stages of HD (Niccolini & Politis, 2014).

The group comparison of all cognitive and brain volumetric variables obtained in our study revealed significant differences between the groups, with the exception of the globus pallidus and the socio‐emotional cognitive test part A. The pallidum has been less well investigated in HD compared to other brain structures (van den Bogaard et al., 2011). However, Rosas et al. (2003) observed significant volume reductions in the pallidum in individuals in the early to mid‐stages of HD. As for the socio‐emotional cognitive test part A, a previous study found that the brain region involved in the feeling of envy is preserved in HD patients (Baez et al., 2016), which may explain the negative findings in our study. Interestingly, we observed that female participants in the patient group and all members of the family group had similar scores in this test. A voxel‐based morphometry (VBM) study identified the gray matter in the dorsolateral prefrontal cortex and superior temporal gyrus as the brain regions specifically related with dispositional (i.e., not episodic) envy (Xiang et al., 2017). Another VBM study in patients with behavioral variant frontotemporal dementia and patients with Alzheimer's disease reported envy to be associated with the anterior cingulate cortex (Santamaría‐García et al., 2017). Liu, Zubieta, and Heitzeg (2012) observed sex differences in these areas that might partly explain the differences in the results of the socio‐emotional cognitive test part A in our study.

Interestingly, although the PVS count and volume in the basal ganglia adjusted by head size did not differ significantly between family and patient groups, the visual scores in this region were significantly higher in patients compared with first‐degree relatives. A previous analysis of computational versus visual neuroradiological assessments of PVS concluded that while computational measurements are least susceptible to be influenced by the overall burden of pathology in the MRI scan, neuroradiological scores capture better the full flavor of pathological versus normal features (Gonzalez‐Castro et al., 2017). Contrary to our hypothesis, PVS visual ratings in all regions were negatively associated with fluid intelligence in the patient group. Studies in nondemented individuals have not ascertained an association between PVS and cognition (Huijts et al., 2014; Hurford et al., 2014; Zhu et al., 2010). Therefore, the lack of association of the PVS burden in the family group is in line with previous findings. Our study is the first to explore the putative role and prevalence of PVS in HD, and our result needs to be confirmed in larger cohorts prior to deriving any conclusions. It is known that mutant Huntingtin forms aggregates that interact with other proteins in neurons causing cell dysfunction by different mechanisms, such as caspase activation, mitochondrial dysfunction, and increased sensitivity to excitotoxicity (Pidgeon & Rickards, 2013). PVS not only contain interstitial and cerebrospinal fluids but they also have a constant flux of macrophages, which are white blood cell of the immune system, and contain vasoactive neuropeptides. The latter, in addition to regulating blood pressure and heart rate, have an integral role in controlling microglia. Some of the mechanisms that cause cell dysfunction, and which are triggered by mutant Huntingtin may cause neuroinflammation. Interestingly, it has been described that perivascular macrophages tend to accumulate during neuroinflammation and cause dilation of the PVS (Pantoni, 2010).

### 4.2 Strengths and weaknesses

To the best of our knowledge, this is the first study to investigate correlates between performance on the Raven matrices test with neuroimaging markers in HD. Although the Raven test provides a nonverbal estimate of fluid intelligence and is often included in research on patients with cognitive deficits (Bilker et al., 2012), fluid intelligence is not a cognitive parameter usually considered in HD. The use of brain structural measures in clinical trials depends on having established an association with a clinically meaningful measure of the disease progression (Tabrizi et al., 2011). Our results will hopefully motivate further studies of the Raven matrices test as a potential cognitive marker for HD. Another strength of our study is that it examines a South American population of a region with high incidence of HD. It is important to research the disease in different ethnic groups as HD is a genetic disease, which varies in incidence across the world, and differences in genetic background and demographics could account for variations in disease prevalence between populations over time (Kay, Hayden, & Leavitt, 2017). The image processing and analysis can be considered another strength of this study. Analyzing and segmenting the MRI scans was done automatically, and results were double‐checked afterwards and edited if necessary to ensure correctness. All measurements were done blind to any other information in order to reduce the risk of observer bias.

A weakness of our study was the lack of information regarding the time from onset of the symptoms in the patient group. This affects the analyses of the results, as patients in the manifest stage of the disease often perform worse on cognitive tests and some cognitive tests are susceptible to floor effects (Ruocco, Bonilha, Li, Lopes‐Cendes, & Cendes, 2008). Another limitation of the study is the absence of genetic information from the first degree relatives. Given our relatively small sample size and the ethnic‐geographical characteristics of our cohort (i.e., from a specific rural region in a South American country), it would be speculative to conclude on the influence of the genetic data in the associations found. Our focus is to evaluate familial vulnerability versus overt disease, adding information to the already known biological (Markianos et al., 2008), clinical (Dorsey, 2012) and cognitive (Baez et al., 2015; Giordani et al., 1995; Kargieman et al., 2014) factors that differentiate both groups. Recent findings of region‐specific atrophies in subcortical structures in premanifest HD underscore the validity of our neuroimaging findings (Tang et al., 2019).

## CONFLICT OF INTEREST

The authors have no conflict of interest to disclose.

## Data Availability

The data that support the findings of this study are available on request from the corresponding author with the permission of the investigators from the following institutions: Universidad Autónoma del Caribe, Universidad de Los Andes, Universidad Favaloro and Strathclyde University. Restrictions apply to the availability of these data, which were used under a collaboration agreement signed by the investigators from the aforementioned institutions with the corresponding author from The University of Edinburgh for the purpose of this study. The imaging data are not publicly available due to privacy and ethical restrictions.

## References

[brb31343-bib-0001] Aylward, E. H. (2014). Magnetic resonance imaging striatal volumes: A biomarker for clinical trials in Huntington's disease. Movement Disorders, 29, 1429–1433. 10.1002/mds.26013 25164586 PMC4479278

[brb31343-bib-0002] Aylward, E. H. , Harrington, D. L. , Mills, J. A. , Nopoulos, P. C. , Ross, C. A. , Long, J. D. , … Paulsen, J. S. (2013). Regional atrophy associated with cognitive and motor function in prodromal Huntington disease. Journal of Huntington's Disease, 2, 477–489. 10.3233/JHD-130076 PMC4412155 25062732

[brb31343-bib-0003] Baez, S. , Herrera, E. , Gershanik, O. , Garcia, A. M. , Bocanegra, Y. , Kargieman, L. , … Ibañez, A. (2015). Impairments in negative emotion recognition and empathy for pain in Huntigton's disease families. Neuropsychologia, 68, 158–167. 25582408 10.1016/j.neuropsychologia.2015.01.012

[brb31343-bib-0004] Baez, S. , Pino, M. , Berrio, M. , Santamaria‐Garcia, H. , Sedeno, L. , Garcia, A. M. , … Ibañez, A. (2018). Corticostriatal signatures of schadenfreude: Evidence from Huntington's disease. Journal of Neurology, Neurosurgery and Psychiatry, 89, 112–116. 10.1136/jnnp-2017-316055 28765320

[brb31343-bib-0005] Baez, S. , Santamaria‐Garcia, H. , Orozco, J. , Fittipaldi, S. , Garcia, A. M. , Pino, M. , & Ibañez, A. (2016). Your misery is no longer my pleasure: Reduced schadenfreude in Huntington's disease families. Cortex, 83, 78–85. 10.1016/j.cortex.2016.07.009 27498039

[brb31343-bib-0006] Ballerini, L. , Lovreglio, R. , Valdés Hernández, M. C. , Ramírez, J. , MacIntosh, B. J. , Black, S. E. , & Wardlaw, J. M. (2018). Perivascular spaces segmentation in brain MRI using optimal 3D filtering. Science Reports, 8, 2132. 10.1038/s41598-018-19781-5 PMC5794857 29391404

[brb31343-bib-0007] Berezuk, C. , Ramirez, J. , Gao, F. , Scott, C. J. , Huroy, M. , Swartz, R. H. , … Boulos, M. I. (2015). Virchow‐Robin spaces: Correlations with polysomnography‐derived sleep parameters. Sleep, 38(6), 853–858. 10.5665/sleep.4726 26163465 PMC4434551

[brb31343-bib-0008] Bilker, W. B. , Hansen, J. A. , Brensinger, C. M. , Richard, J. , Gur, R. E. , & Gur, R. C. (2012). Development of abbreviated nine‐item forms of the Raven's standard progressive matrices test. Assessment, 19, 354–369. 10.1177/1073191112446655 22605785 PMC4410094

[brb31343-bib-0009] Bonelli, R. M. , & Cummings, J. L. (2007). Frontal‐subcortical circuitry and behavior. Dialogues in Clinical Neuroscience, 9, 141–151. 17726913 10.31887/DCNS.2007.9.2/rbonelli PMC3181854

[brb31343-bib-0010] Cheung, C. C. , Lee, T. M. , Yip, J. T. , King, K. E. , & Li, L. S. (2006). The differential effects of thalamus and basal ganglia on facial emotion recognition. Brain and Cognition, 61, 262–268. 10.1016/j.bandc.2006.01.008 16540222

[brb31343-bib-0011] Ciarmiello, A. , Giovacchini, G. , Giovannini, E. , Lazzeri, P. , Borso, E. , Mannironi, A. , & Mansi, L. (2017). Molecular imaging of Huntington's disease. Journal of Cellular Physiology, 232, 1988–1993. 10.1002/jcp.25666 27791273

[brb31343-bib-0012] Coppen, E. M. , Jacobs, M. , van den Berg‐Huysmans, A. A. , van der Grond, J. , & Roos, R. A. C. (2018). Grey matter volume loss is associated with specific clinical motor signs in Huntington's disease. Parkinsonism & Related Disorders, 46, 56–61. 10.1016/j.parkreldis.2017.11.001 29128164

[brb31343-bib-0013] Dorsey, E. (2012). Characterization of a large group of individuals with Huntington's disease and their relatives enrolled in the COHORT study. PLoS ONE, 7(2), e29522. 22359536 10.1371/journal.pone.0029522 PMC3281013

[brb31343-bib-0014] Duperron, M. G. , Tzourio, C. , Sargurupremraj, M. , Mazoyer, B. , Soumaré, A. , Schilling, S. , … Debette, S. (2018). Burden of dilated perivascular spaces, an emerging marker of cerebral small vessel disease, is highly heritable. Stroke, 49(2), 282–287. 10.1161/STROKEAHA.117.019309 29311265

[brb31343-bib-0015] Francis, F. , Ballerini, L. , & Wardlaw, J. M. (2019). Perivascular spaces and their associations with risk factors, clinical disorders and neuroimaging features: A systematic review and meta‐analysis. International Journal of Stroke. 10.1177/1747493019830321 30762496

[brb31343-bib-0016] Georgiou‐Karistianis, N. , Scahill, R. , Tabrizi, S. J. , Squitieri, F. , & Aylward, E. (2013). Structural MRI in Huntington's disease and recommendations for its potential use in clinical trials. Neuroscience and Biobehavioral Reviews, 37, 480–490. 10.1016/j.neubiorev.2013.01.022 23376047

[brb31343-bib-0017] Giordani, B. , Berent, S. , Boivin, M. J. , Penney, J. B. , Lehtinen, S. , Markel, D. S. , … Young, A. B. (1995). Longitudinal neuropsychological and genetic linkage analysis of persons at risk for Huntington's disease. Archives of Neurology, 52(1), 59–64. 10.1001/archneur.1995.00540250063014 7826277

[brb31343-bib-0018] Gonzalez‐Castro, V. , Valdés Hernández, M. D. C. , Chappell, F. M. , Armitage, P. A. , Makin, S. , & Wardlaw, J. M. (2017). Reliability of an automatic classifier for brain enlarged perivascular spaces burden and comparison with human performance. Clinical Science, 131(13), 1465–1481. 28468952 10.1042/CS20170051

[brb31343-bib-0019] Hobbs, N. Z. , Henley, S. M. , Ridgway, G. R. , Wild, E. J. , Barker, R. A. , Scahill, R. I. , … Tabrizi, S. J. (2010). The progression of regional atrophy in premanifest and early Huntington's disease: A longitudinal voxel‐based morphometry study. Journal of Neurology, Neurosurgery and Psychiatry, 81, 756–763. 10.1136/jnnp.2009.190702 19955112

[brb31343-bib-0020] Huijts, M. , Duits, A. , Staals, J. , Kroon, A. A. , de Leuw, P. W. , & van Oostenbrugge, R. J. (2014). Basal ganglia enlarged perivascular spaces are linked to cognitive function in patients with cerebral small vessel disease. Current Neurovascular Research, 11, 136–141. 24606607 10.2174/1567202611666140310102248

[brb31343-bib-0021] Hurford, R. , Charidimou, A. , Fox, Z. , Cipolotti, L. , Jager, R. , & Werring, D. J. (2014). MRI‐visible perivascular spaces: Relationship to cognition and small vessel disease MRI markers in ischaemic stroke and TIA. Journal of Neurology, Neurosurgery and Psychiatry, 85, 522–525. 10.1136/jnnp-2013-305815 PMC3995332 24249785

[brb31343-bib-0022] Jurgens, C. K. , van de Wiel, L. , van Es, A. C. , Grimbergen, Y. M. , Witjes‐Ane, M. N. , van der Grond, J. , … Roos, R. A. (2008). Basal ganglia volume and clinical correlates in 'preclinical' Huntington's disease. Journal of Neurology, 255, 1785–1791. 10.1007/s00415-008-0050-4 19156490

[brb31343-bib-0023] Kargieman, L. , Herrera, E. , Baez, S. , García, A. M. , Dottori, M. , Gelormini, C. , … Ibáñez, A. (2014). Motor‐language coupling in Huntington's disease families. Frontiers in Aging Neuroscience, 6, 122. 24971062 10.3389/fnagi.2014.00122 PMC4054328

[brb31343-bib-0024] Kassubek, J. , Juengling, F. D. , Ecker, D. , & Landwehrmeyer, G. B. (2005). Thalamic atrophy in Huntington's disease co‐varies with cognitive performance: A morphometric MRI analysis. Cerebral Cortex, 15, 846–853. 10.1093/cercor/bhh185 15459079

[brb31343-bib-0025] Kay, C. , Hayden, M. R. , & Leavitt, B. R. (2017). Epidemiology of Huntington disease. Handbook of Clinical Neurology, 144, 31–46. 28947124 10.1016/B978-0-12-801893-4.00003-1

[brb31343-bib-0026] Kim, S. D. , & Fung, V. S. (2014). An update on Huntington's disease: From the gene to the clinic. Current Opinion in Neurology, 27(4), 477–483. 24978638 10.1097/WCO.0000000000000116

[brb31343-bib-0027] Kong, H. (2010). Root of Thought. Reflections on Neuroscience. P212 Ebook iUniverse, ISBN 9781450222686.

[brb31343-bib-0028] Lawrence, A. D. , Sahakian, B. J. , & Robbins, T. W. (1998). Cognitive functions and corticostriatal circuits: Insights from Huntington's disease. Trends in Cognitive Sciences, 2(10), 379–388. 10.1016/S1364-6613(98)01231-5 21227253

[brb31343-bib-0029] Liebermann, D. , Ostendorf, F. , Kopp, U. A. , Kraft, A. , Bohner, G. , Nabavi, D. G. , … Ploner, C. J. (2013). Subjective cognitive‐affective status following thalamic stroke. Journal of Neurology, 260(2), 386–396. 10.1007/s00415-012-6635-y 22854887

[brb31343-bib-0030] Liu, J. , Zubieta, J.‐K. , & Heitzeg, M. (2012). Sex differences in anterior cingulate cortex activation during impulse inhibition and behavioural correlates. Psychiatry Research, 201(1), 54–62. 22285718 10.1016/j.pscychresns.2011.05.008 PMC3289751

[brb31343-bib-0031] Markianos, M. , Panas, M. , Kalfakis, N. , & Vassilopoulos, D. (2008). Low plasma total cholesterol in patients with Huntington's disease and first‐degree relatives. Molecular Genetics and Metabolism, 93(3), 341–346. 10.1016/j.ymgme.2007.10.002 18006350

[brb31343-bib-0032] McColgan, P. , & Tabrizi, S. J. (2018). Huntington's disease: A clinical review. European Journal of Neurology, 25, 24–34. 10.1111/ene.13413 28817209

[brb31343-bib-0033] Mestre, H. , Kostrikov, S. , Mehta, R. I. , & Nedergaard, M. (2017). Perivascular spaces, glymphatic dysfunction, and small vessel disease. Clinical Science (Lond), 131(17), 2257–2274. 10.1042/CS20160381 PMC5567781 28798076

[brb31343-bib-0034] Misiura, M. B. , Lourens, S. , Calhoun, V. D. , Long, J. , Bockholt, J. , Johnson, H. , … Fall, E. (2017). Cognitive control, learning, and clinical motor ratings are most highly associated with Basal Ganglia Brain Volumes in the Premanifest Huntington's Disease Phenotype. Journal of the International Neuropsychological Society, 23, 159–170. 10.1017/S1355617716001132 28205498 PMC5803794

[brb31343-bib-0035] Mo, C. , Hannan, A. J. , & Renoir, T. (2015). Environmental factors as modulators of neurodegeneration: Insights from gene‐environment interactions in Huntington's disease. Neuroscience and Biobehavioral Reviews, 52, 178–192. 10.1016/j.neubiorev.2015.03.003 25770041

[brb31343-bib-0036] Myers, R. H. , Sax, D. S. , Koroshetz, W. J. , Mastromauro, C. , Cupples, L. A. , Kiely, D. K. , … Bird, E. D. (1991). Factors associated with slow progression in Huntington's disease. Archives of Neurology, 48, 800–804. 10.1001/archneur.1991.00530200036015 1832854

[brb31343-bib-0037] Nasreddine, Z. S. , Phillips, N. A. , Bedirian, V. , Charbonneau, S. , Whitehead, V. , Collin, I. , … Chertkow, H. (2005). The montreal cognitive assessment, MoCA: A brief screening tool for mild cognitive impairment. Journal of the American Geriatrics Society, 53, 695–699. 10.1111/j.1532-5415.2005.53221.x 15817019

[brb31343-bib-0038] Niccolini, F. , & Politis, M. (2014). Neuroimaging in Huntington's disease. World Journal of Radiology, 6, 301–312. 10.4329/wjr.v6.i6.301 24976932 PMC4072816

[brb31343-bib-0040] Pantoni, L. (2010). Cerebral small vessel disease: From pathogenesis and clinical characteristics to therapeutic challenges. The Lancet Neurology, 9(7), 689–701. 20610345 10.1016/S1474-4422(10)70104-6

[brb31343-bib-0041] Pessoa, L. (2017). A network model of the emotional brain. Trends in Cognitive Sciences, 21(5), 357–371. 10.1016/j.tics.2017.03.002 28363681 PMC5534266

[brb31343-bib-0042] Pidgeon, C. , & Rickards, H. (2013). The pathophysiology and pharmacological treatment of Huntington disease. Behavioural Neurology, 26, 245–253. 10.1155/2013/705373 22713409 PMC5214454

[brb31343-bib-0043] Potter, G. M. , Chappell, F. M. , Morris, Z. , & Wardlaw, J. M. (2015). Cerebral perivascular spaces visible on magnetic resonance imaging: Development of a qualitative rating scale and its observer reliability. Cerebrovascular Disease, 39(3–4), 224–231. 10.1159/000375153 PMC4386144 25823458

[brb31343-bib-0044] Ramirez, J. , Berezuk, C. , McNeely, A. A. , Scott, C. J. , Gao, F. , & Black, S. E. (2015). Visible Virchow‐Robin spaces on magnetic resonance imaging of Alzheimer's disease patients and normal elderly from the Sunnybrook Dementia Study. Journal of Alzheimer's Disease, 43(2), 415–424. 10.3233/JAD-132528 25096616

[brb31343-bib-0046] Rosas, H. D. , Koroshetz, W. J. , Chen, Y. I. , Skeuse, C. , Vangel, M. , Cudkowicz, M. E. , … Goldstein, J. M. (2003). Evidence for more widespread cerebral pathology in early HD: An MRI‐based morphometric analysis. Neurology, 60, 1615–1620. 10.1212/01.WNL.0000065888.88988.6E 12771251

[brb31343-bib-0047] Ruocco, H. H. , Bonilha, L. , Li, L. M. , Lopes‐Cendes, I. , & Cendes, F. (2008). Longitudinal analysis of regional grey matter loss in Huntington disease: Effects of the length of the expanded CAG repeat. Journal of Neurology, Neurosurgery and Psychiatry, 79, 130–135. 10.1136/jnnp.2007.116244 17615168

[brb31343-bib-0048] Santamaría‐García, H. , Baez, S. , Reyes, P. , Santamaría‐García, J. A. , Santacruz‐Escudero, J. M. , Matallana, D. , … Ibáñez, A. (2017). A lesion model of envy and *Schadenfreude*: Legal, deservingness and moral dimensions as revealed by neurodegeneration. Brain, 140(12), 3357–3377. 29112719 10.1093/brain/awx269 PMC5841144

[brb31343-bib-0049] Scahill, R. I. , Andre, R. , Tabrizi, S. J. , & Aylward, E. H. (2017). Structural imaging in premanifest and manifest Huntington disease. Handbook of Clinical Neurology, 144, 247–261. 28947121 10.1016/B978-0-12-801893-4.00020-1

[brb31343-bib-0050] Shoulson, I. , & Fahn, S. (1979). Huntington's disease: Clinical care and evaluation. Neurology, 29, 1–3. 154626 10.1212/wnl.29.1.1

[brb31343-bib-0051] Siesling, S. , van Vugt, J. P. , Zwinderman, K. A. , Kieburtz, K. , & Roos, R. A. (1998). Unified Huntington's disease rating scale: A follow up. Movement Disorders, 13, 915–919. 10.1002/mds.870130609 9827615

[brb31343-bib-0052] Sun, Y. , Yu, H. , Chen, J. , Liang, J. , Lu, L. , Zhou, X. , & Shi, J. (2015). Neural substrates and behavioural profiles of romantic jealousy and its temporal dynamics. Science Reports, 6, 27469. 10.1038/srep27469 PMC4895349 27273024

[brb31343-bib-0053] Tabrizi, S. J. , Langbehn, D. R. , Leavitt, B. R. , Roos, R. A. , Durr, A. , Craufurd, D. , … Stout, J. C. (2009). Biological and clinical manifestations of Huntington's disease in the longitudinal TRACK‐HD study: Cross‐sectional analysis of baseline data. The Lancet Neurology, 8, 791–801. 10.1016/S1474-4422(09)70170-X 19646924 PMC3725974

[brb31343-bib-0054] Tabrizi, S. J. , Scahill, R. I. , Durr, A. , Roos, R. A. , Leavitt, B. R. , Jones, R. , … Stout, J. C. (2011). Biological and clinical changes in premanifest and early stage Huntington's disease in the TRACK‐HD study: The 12‐month longitudinal analysis. The Lancet Neurology, 10, 31–42. 10.1016/S1474-4422(10)70276-3 21130037

[brb31343-bib-0055] Tabrizi, S. J. , Scahill, R. I. , Owen, G. , Durr, A. , Leavitt, B. R. , Roos, R. A. , … Langbehn, D. R. (2013). Predictors of phenotypic progression and disease onset in premanifest and early‐stage Huntington's disease in the TRACK‐HD study: Analysis of 36‐month observational data. The Lancet Neurology, 12, 637–649. 10.1016/S1474-4422(13)70088-7 23664844

[brb31343-bib-0056] Tang, X. , Ross, C. A. , Johnson, H. , Paulsen, J. S. , Younes, L. , Albin, R. L. , … Miller, M. I. (2019). Regional subcortical shape analysis in premanifest Huntington's disease. Human Brain Mapping, 40(5), 1419–1433. 10.1002/hbm.24456 30376191 PMC6420821

[brb31343-bib-0057] Torralva, T. , Roca, M. , Gleichgerrcht, E. , Lopez, P. , & Manes, F. (2009). INECO Frontal Screening (IFS): A brief, sensitive, and specific tool to assess executive functions in dementia. Journal of the International Neuropsychological Society, 15, 777–786. 19635178 10.1017/S1355617709990415

[brb31343-bib-0058] Valdés Hernández, M. C. , Armitage, P. A. , Thrippleton, M. J. , Chappell, F. , Sandeman, E. , Muñoz Maniega, S. , … Wardlaw, J. M. (2015). Rationale, design and methodology of the image analysis protocol for studies of patients with cerebral small vessel disease and mild stroke. Brain and Behavior, 5(12), e00415 10.1002/brb3.415 26807340 PMC4714639

[brb31343-bib-0059] van den Bogaard, S. J. , Dumas, E. M. , Acharya, T. P. , Johnson, H. , Langbehn, D. R. , Scahill, R. I. , … Roos, R. A. (2011). Early atrophy of pallidum and accumbens nucleus in Huntington's disease. Journal of Neurology, 258, 412–420. 10.1007/s00415-010-5768-0 20936300 PMC3112014

[brb31343-bib-0060] Wardlaw, J. M. , Smith, E. E. , Biessels, G. J. , Cordonnier, C. , Fazekas, F. , Frayne, R. , … Dichgans, M. (2013). Neuroimaging standards for research into small vessel disease and its contribution to ageing and neurodegeneration. The Lancet Neurology, 12(8), 822–838. 10.1016/S1474-4422(13)70124-8 23867200 PMC3714437

[brb31343-bib-0061] Wilkos, E. , Brown, T. J. , Slawinska, K. , & Kucharska, K. A. (2015). Social cognitive and neurocognitive deficits in inpatients with unilateral thalamic lesions ‐ pilot study. Neuropsychiatric Disease and Treatment, 11, 1031–1038. 25914535 10.2147/NDT.S78037 PMC4401357

[brb31343-bib-0062] Xiang, Y. , Zhao, S. , Wang, H. , Wu, Q. , Kong, F. , & Mo, L. (2017). Examining brain structures associated with dispositional envy and the mediation role of emotional intelligence. Science Reports, 7, 39947. 10.1038/srep39947 PMC5296859 28176785

[brb31343-bib-0063] Zhu, Y. C. , Dufouil, C. , Soumare, A. , Mazoyer, B. , Chabriat, H. , & Tzourio, C. (2010). High degree of dilated Virchow‐Robin spaces on MRI is associated with increased risk of dementia. Journal of Alzheimer's Disease, 22, 663–672. 10.3233/JAD-2010-100378 20847444

