# Supplementary material for: Structural neuroimaging differentiates vulnerability from disease manifestation in colombian families with Huntington's disease
Source: Brain Behav. 2019 Jul 5;9(8):e01343. doi: 10.1002/brb3.1343 (PMC6710228; doi:10.1002/brb3.1343)
Supplement: Supplementary file 1 [file BRB3-9-e01343-s001.docx]

**Structural Neuroimaging Differential Indicators of Vulnerability from Overt Disease in Colombian Huntington’s Disease Families**

Maria del C. Valdés Hernández, Janna Abu-Hussain, Xinyi Qiu, Josef Priller, Mario Parra Rodríguez, Mariana Pino, Sandra Báez, Agustín Ibáñez

**Supplementary Material**

Table S1. Descriptive statistics of the variables involved in the analyses. Numeric results are displayed up to two significant figures.

|  | | **HD Patients (mean+/-SD)** | | **First-Degree Relatives**  **(mean +/-SD)** | | **Total** |
| --- | --- | --- | --- | --- | --- | --- |
|  |  | **Male** | **Female** | **Male** | **Female** |  |
| **Demographic Variables (years)** | Age | 52.14 ± 7.58 | 40.38 ± 7.39 | 32.67 ± 11.06 | 24.50 ± 6.17 | 36.76 ± 12.86 |
|  | Education | 8.86 ± 2.035 | 9.50 ± 4.071 | 13.67 ± 2.89 | 12.5 ± 2.014 | 10.97 ± 3.26 |
| **Imaging Volumetric Variables (% in ICV)** | NAWM | 35.61 ± 1.90 | 35.78 ± 1.57 | 38.39 ± 2.17 | 37.97 ± 1.57 | 36.80 ± 2.017 |
|  | CSF | 30.35 ± 2.70 | 26.51 ± 2.28 | 20.34 ± 1.72 | 19.46 ± 2.58 | 24.29 ± 5.17 |
|  | Left Caudate Nucleus | 0.09 ± 0.025 | 0.12 ± 0.049 | 0.17 ± 0.047 | 0.21 ± 0.031 | 0.15 ± 0.061 |
|  | Right Caudate Nucleus | 0.054 ± 0.046 | 0.13 ± 0.049 | 0.19 ± 0.051 | 0.22 ± 0.031 | 0.15 ± 0.078 |
|  | Left Putamen | 0.055 ± 0.021 | 0.093 ± 0.022 | 0.14 ± 0.060 | 0.14 ± 0.029 | 0.11 ± 0.046 |
|  | Right Putamen | 0.055 ± 0.016 | 0.086 ± 0.020 | 0.17 ± 0.045 | 0.15 ± 0.038 | 0.11 ± 0.053 |
|  | Left Globus Pallidus | 0.004 ± 0.0029 | 0.0088 ± 0.0061 | 0.0078 ± 0.0061 | 0.0067 ± 0.0032 | 0.0068 ± 0.0041 |
|  | Right Globus Pallidus | 0.0043 ± 0.0027 | 0.006 ± 0.0037 | 0.011 ± 0.0057 | 0.0066 ± 0.0043 | 0.0063 ± 0.0041 |
|  | Left Thalamus | 0.14 ± 0.028 | 0.18 ± 0.015 | 0.19 ± 0.026 | 0.22 ± 0.032 | 0.18 ± 0.040 |
|  | Right Thalamus | 0.15 ± 0.014 | 0.18 ± 0.019 | 0.22 ± 0.029 | 0.22 ± 0.026 | 0.19 ± 0.036 |
|  | BGPVS | 0.023 ± 0.012 | 0.037 ± 0.020 | 0.032 ± 0.0041 | 0.017 ± 0.0060 | 0.026 ± 0.015 |
| **PVS visual scores (Potter scale)** | Basal Ganglia | 2 (1) | 1 (1) | 1 (0) | 1 (0) | 1 (1) |
|  | Centrum Semiovale | 2 (2) | 1 (1) | 1 (0) | 1 (1) | 1 (1) |
|  | Midbrain | 1 (1) | 0 (1) | 1 (0) | 0 (0) | 0 (1) |
| **Functional, Cognitive and Socio-emotional Assessment Scores (arbitrary units)** | Myer’s FS | 75.71 ± 5.34 | 77.50 ± 19.82 | 100.00 ± 0 | 99.00 ± 3.16 | 87.59 ± 15.51 |
|  | IFS_Total | 11.71 ± 6.10 | 17.00 ± 4.78 | 25.00 ± 1.73 | 21.40 ± 2.76 | 18.17 ± 6.00 |
|  | MOCA Total | 15.86 ± 4.74 | 18.13 ± 4.67 | 27.67 ± 0.58 | 25.70 ± 3.13 | 21.24 ± 5.92 |
|  | Raven Matrices | 3.86 ± 1.34 | 5.38 ± 5.73 | 9.67 ± 8.083 | 12.80 ± 6.39 | 7.97 ± 6.46 |
|  | Soc. Emot. Part A Total | 25.00 ± 22.61 | 35.63 ± 17.71 | 33.67 ± 0.58 | 37.70 ± 19.42 | 34.41 ± 18.95 |
|  | Soc. Emot. Part B Total | 44.57 ± 18.38 | 44.25 ± 21.26 | 37.00 ± 5.20 | 19.30 ± 11.28 | 33.90 ± 19.50 |


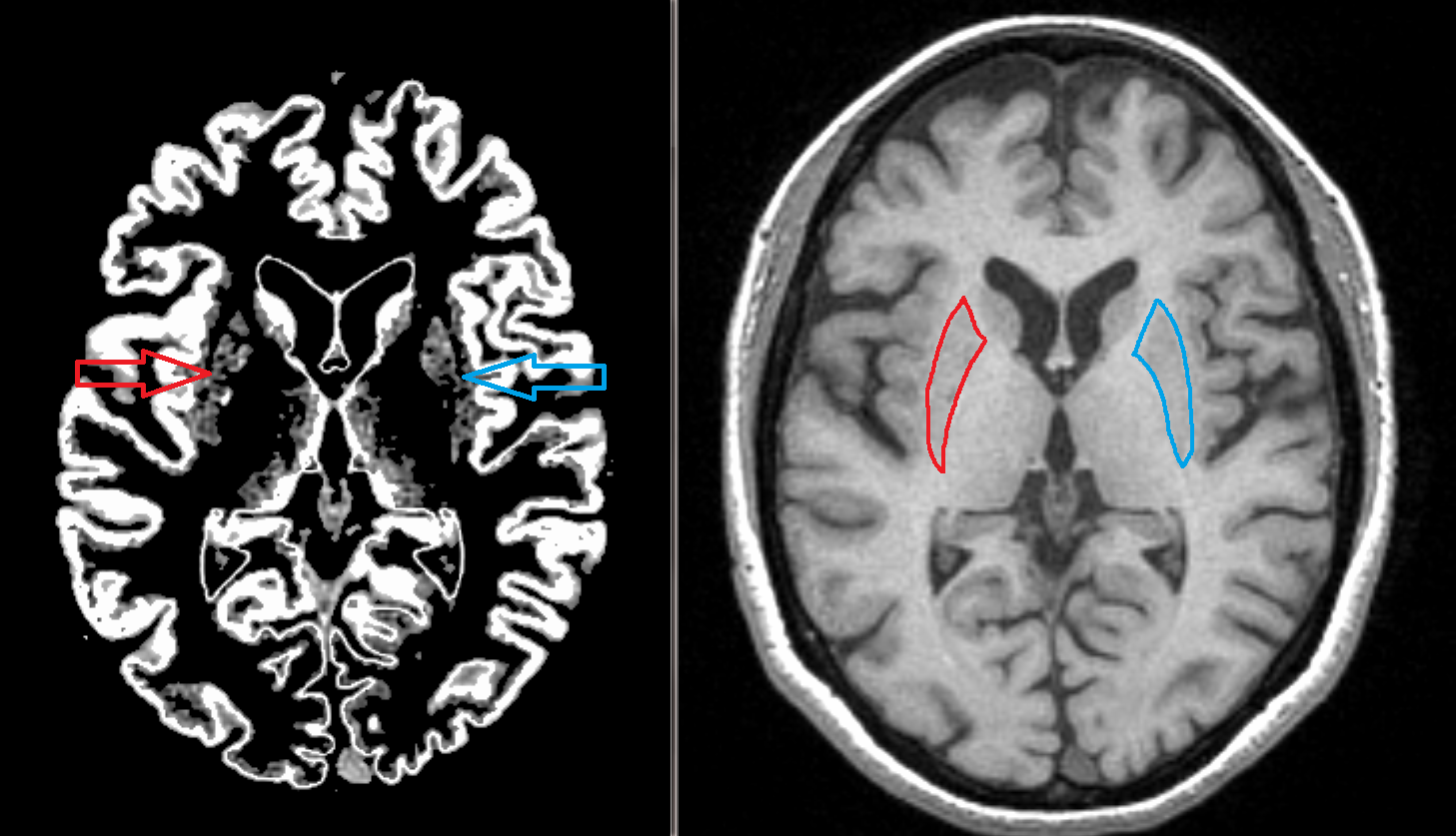


Figure S1. Illustration of the conceptual difference between the subcortical structural volumes and grey matter volume in the subcortical structures (i.e. the measurements evaluated in this study). The right panel shows a T1-weighted MR axial slice of a study participant with the boundaries of the left and right putamen delineated in blue and red respectively (radiological orientation). The left panel shows the result of the automatic segmentation of the grey matter, with arrows indicating the grey matter volume of the left and right putamen.

Table S2. Numeric results of the mean differences between the subcortical measurements (i.e. between the total volume of the structure and the volume of the grey matter tissue on the structure) and their confidence intervals. Values are expressed as percentage with respect to the mean volume of each structure.

| Measurement | Mean difference +/- 2SD between the raw volumes (%) | Mean difference [+/- 2SD] after being adjusted by intracranial volume (%) |
| --- | --- | --- |
| Left caudate | 18.47 +/- 15.26 | 17.43 +/- 15.17 |
| Right caudate | 19.85 +/- 16.58 | 20.79 +/- 15.10 |
| Left globus pallidus | 100.29 +/- 153.31 | 117.47 +/- 165.70 |
| Right globus pallidus | 99.60 +/- 138.24 | 108.09 +/- 144.49 |
| Left putamen | 23.29 +/- 33.90 | 22.42 +/- 33.80 |
| Right putamen | 20.36 +/- 36.01 | 19.63 +/- 36.50 |
| Left thalamus | 21.13 +/- 20.42 | 20.26 +/- 21.25 |
| Right thalamus | 18.77 +/- 17.52 | 17.83 +/- 17.98 |


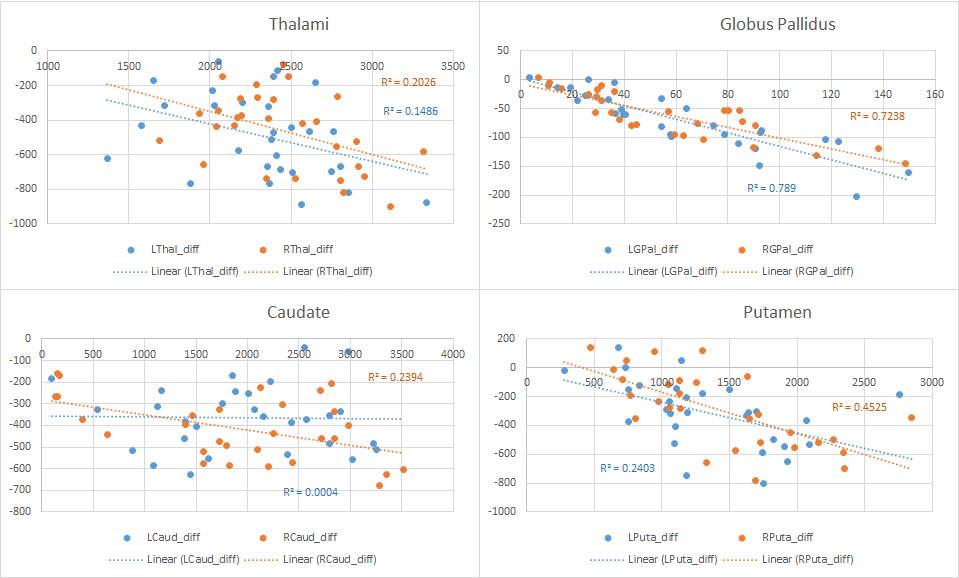


Figure S2. Differences (in mm^3^) of the raw volumetric measurements of the grey matter in the subcortical structures analysed and of the total subcortical structural volumes (y axis) versus their average values (x axis also in mm^3^). The trends in the volumetric differences with respect to their average values is also shown. Orange measurements correspond to the structures on the right cerebral hemisphere and blue measurements correspond to the structures on the left.
